# Successful Treatment of Anti‐Jo‐1 Positive Inflammatory Myopathy With Tofacitinib

**DOI:** 10.1155/crrh/6478835

**Published:** 2026-06-03

**Authors:** Janis Timsans, Anna-Mari Hokkanen, Joonas Rautavaara, Anne Kerola, Sanna Huovinen, Markku Kauppi

**Affiliations:** ^1^ Faculty of Medicine and Health Technology, Tampere University, Tampere, Finland, uta.fi; ^2^ Department of Rheumatology, Päijät-Häme Central Hospital, Wellbeing Services County of Päijät-Häme, Lahti, Finland, phsotey.fi; ^3^ University of Helsinki, Helsinki, Finland, helsinki.fi; ^4^ Department of Pathology, Fimlab Laboratories, Tampere University Hospital, Tampere, Finland, pshp.fi

## Abstract

Idiopathic inflammatory myopathies are, in some cases, refractory to all guideline‐based treatments, posing a very difficult challenge in clinical practice. Janus kinase inhibitors are a rather new class of disease‐modifying antirheumatic drugs that are approved for the treatment of rheumatoid arthritis, spondyloarthritis, and psoriatic arthritis, but have been used as an off‐label treatment only in a very limited number of cases in inflammatory myopathies. In this case report, we present a middle‐aged woman with severe muscle weakness and pain attributed to refractory myositis associated with anti‐Jo‐1 antibody. Despite multiple treatment attempts, which included all guideline‐based immunosuppressants, the patient’s condition remained unresponsive. Initiation of tofacitinib therapy resulted in significant clinical improvement, normalization of creatinine kinase levels, and being possible to discontinue glucocorticoids. Our findings highlight the potential efficacy of tofacitinib in refractory inflammatory myopathies, and to our knowledge, our case is the first one to demonstrate the efficacy of a Janus kinase inhibitor in anti‐Jo‐1 positive myopathy without interstitial lung disease.

## 1. Introduction

Even though there have been tremendous advancements in the treatment of rheumatic disorders lately, idiopathic inflammatory myopathies (IIMs) in some cases do not respond to glucocorticoid‐sparing agents that are supported by current guidelines. Janus kinase inhibitors (JAKis) have been demonstrated to be useful in refractory cases of IIM in some reports, but the evidence is still rather scarce. The objective of this case report is to demonstrate a case where the JAKi tofacitinib was effective in the treatment of refractory myositis associated with anti‐Jo‐1 antibody.

## 2. Case Presentation

**FIGURE 1:**
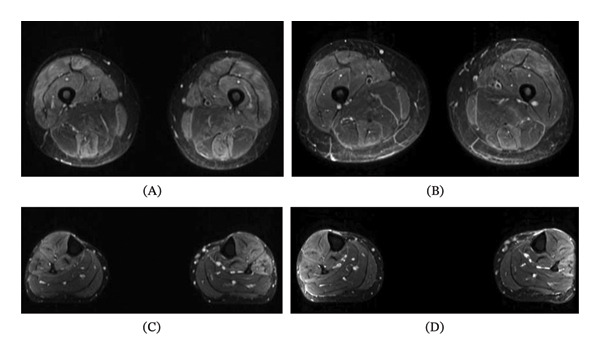
MRI (STIR sequence) findings of the muscles of the lower extremities. (A) MRI of thighs in October 2022 showing edema of the quadriceps femoris, gracilis, and semitendinosus muscles. (B) MRI of thighs in May 2023 showing slight resolution of the edema of the quadriceps femoris, gracilis, and semitendinosus muscles, but no muscle atrophy. (C) MRI of shanks in October 2022 showing edema in the tibialis anterior and peroneus muscles. (D) MRI of shanks in May 2023 showing edema in the tibialis anterior muscles and peroneus muscles with no muscle atrophy.

**FIGURE 2:**
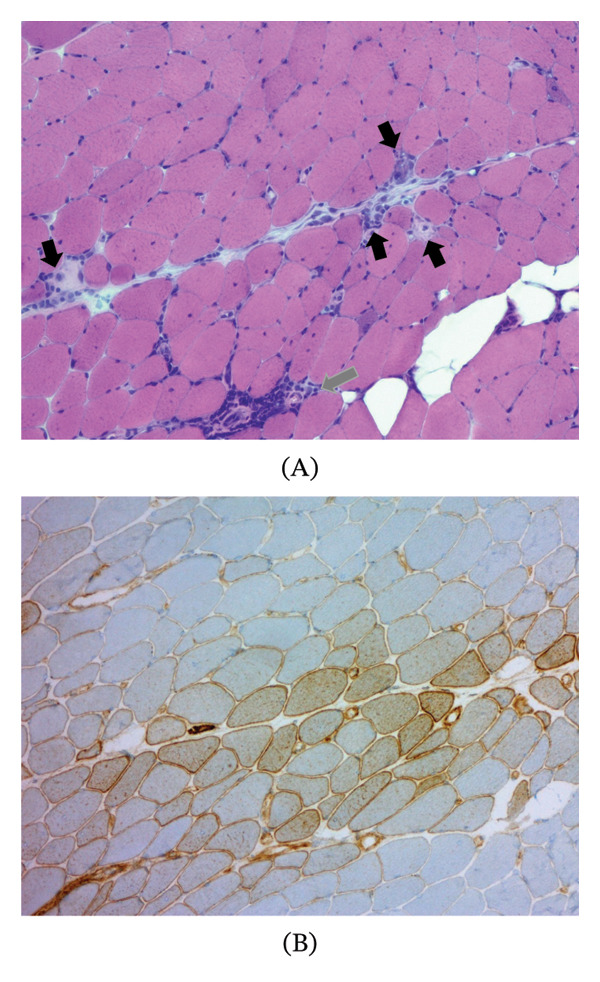
Muscle biopsy findings. (A) Hematoxylin and eosin staining demonstrates perifascicular necrotic fibers (black arrows) and mild perivascular inflammatory infiltrates (gray arrow). (B) Immunohistochemistry for MHC Class I shows perifascicular upregulation.

A middle‐aged white woman, who had previously smoked and had been diagnosed with hypothyreosis, asthma, hypercholesterolemia, and hypertension, experienced proximal muscle weakness and pain since 2019. Creatinine kinase (CK) was markedly elevated (1656 U/L). Findings compatible with IIM were found in both electroneuromyography and MRI (Figures 1A and C). Muscle biopsy revealed inflammatory myopathy with perifascicular necrosis and MHC Class I upregulation in perifascicular regions (Figure 2). These findings are consistent with antisynthetase syndrome (ASyS)–associated myositis [1–3]. Myopathology in ASyS has been increasingly recognized as a distinct entity characterized by perifascicular necrosis rather than perifascicular atrophy, which is typical of dermatomyositis (DM) [4].

Anti‐Jo‐1 and anti‐Ro/SSA antibodies were detected. The patient was diagnosed with ASyS, with inflammatory myopathy as the main manifestation. No clear signs of ILD were detected. No findings of connective tissue diseases were detected. The disease, however, affected the myocardium, leading to an increase in Troponin T levels up to 1362 ng/L, and there were findings of cardiovascular myopathy observed in cardiac MRI. The clinical presentation of the disease was severe, requiring hospitalizations and impairing the daily functioning of the patient to the level where independent staying at patient’s own home was at risk.

The disease was initially treated with high‐dose glucocorticoids (prednisolone 60 mg/day). In summer 2020, the patient received rituximab infusions (1000 mg administered twice, two weeks apart); however, the disease remained active, with CK levels rising to 10,000 U/L, necessitating reintroduction of high‐dose glucocorticoids.

Subsequently, six monthly cyclophosphamide infusions (1000 mg per infusion) were administered, resulting in only slight clinical improvement. Conventional immunosuppressive therapies, including methotrexate, cyclosporine, mycophenolate mofetil, and azathioprine at maximally tolerated doses, were ineffective, with no meaningful impact on CK levels or clinical symptoms.

**FIGURE 3:**
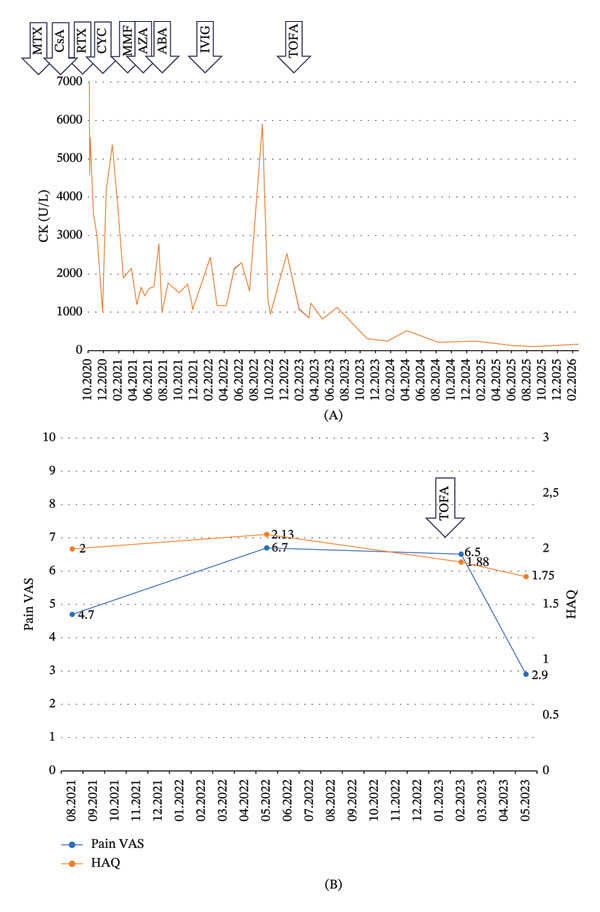
Changes in CK levels and patient‐reported outcome measures. (A) Changes in CK levels. (B) Changes in HAQ score and pain VAS. Arrows indicate the start of a specific medication. CK: creatinine kinase; HAQ: Health assessment questionnaire score; VAS: visual analog scale; MTX: methotrexate; CsA: cyclosporin; RTX: rituximab; MMF: mycophenolate mofetil; AZA: azathioprine; ABA: abatacept; IVIG: intravenous immunoglobulin; TOFA: tofacitinib.

Intravenous abatacept, which has shown efficacy in some studies [5, 6], was initiated (750 mg at Weeks 0, 2, and 4, followed by administration every 4 weeks), but proved ineffective in this case. Intravenous immunoglobulin (IVIG) therapy (three cycles administered 1 month apart, at a dose of 0.4 g/kg/day for three consecutive days) was also ineffective (Figure 3A).

Throughout these treatment attempts, the patient required oral glucocorticoids at doses ranging from 7.5 to 30 mg of prednisolone daily. As a consequence of prolonged moderate‐ to high‐dose glucocorticoid therapy, osteopenia developed. Due to progressive muscle weakness and pain, the patient required a rollator for ambulation.

In January 2023, after discussing the potential risks with the patient, a trial of tofacitinib was initiated at a dose of 11 mg once daily, which led to marked clinical improvement. The patient has now been receiving tofacitinib for more than 3 years and continues to do well. CK levels decreased rapidly during the first months of treatment and have remained within the normal range (Figure 3A). Troponin T levels also declined quickly, indicating resolution of the inflammatory process in the myocardium, and subsequently decreased to a near‐normal value of 26 ng/L.

In 2024, the patient was able to reduce the prednisolone dose to 2.5 mg daily and subsequently discontinue glucocorticoid therapy completely. The patient’s daily functioning improved and pain diminished substantially (Figure 3B), and the need for a walking aid resolved.

Follow‐up MRI of the lower extremity muscles performed in May 2023 (i.e., only a few months after initiation of tofacitinib) demonstrated partial resolution of edema in the quadriceps femoris, gracilis, semitendinosus, tibialis anterior, and peroneal muscles (Figures 1B and D), consistent with treatment efficacy. Importantly, no muscle atrophy was observed, suggesting that the decrease in CK levels reflected reduced disease activity rather than muscle loss, which can sometimes occur in longstanding IIM. Because the patient’s clinical condition improved markedly, no additional MRI examinations were considered necessary after May 2023.

## 3. Discussion

Due to the rarity and heterogeneity of IIMs, treatment guidelines are sparse and varied [7]. The mainstay treatment in the current recommendations is glucocorticoids and facilitating a steroid‐free remission using conventional synthetic DMARDs. As second‐line treatments, rituximab, cyclophosphamide, IVIG, and abatacept are proposed [8]. Some patients, however, do not respond to any of these therapies. JAK inhibitors may be effective, especially in anti‐MDA5 positive DM [9], but evidence in ASyS is scarce.

There have been some promising reports of using JAKis in IIMs [10], but the evidence is regarded as insufficient to include JAKis in the treatment guidelines [9]. Tofacitinib is the most frequently used JAK inhibitor in previously reported cases, which influenced our decision to initiate treatment with this agent in our patient. Tofacitinib has been shown to be effective in the treatment of ILD in a patient with anti–Jo‐1–positive DM *sine* myositis [11] and in a series of nearly 20 ASyS patients, all of whom had ILD [12]. To our knowledge, our case is the first to report the effectiveness of tofacitinib in anti–Jo‐1–positive myopathy without ILD. Upregulation of interferon (IFN)‐regulated genes in IIMs may provide a mechanistic explanation for the efficacy of JAKis, as these agents block IFN signaling through the Janus kinase pathway [13]. However, Type I and Type II IFN pathways appear to be differentially activated across myositis subtypes: expression of Type I IFN–inducible genes is reported to be high in DM, moderate in ASyS, and low in immune‐mediated necrotizing myopathy (IMNM) and inclusion body myositis (IBM). In contrast, expression of Type II IFN–inducible genes is high in DM, IBM, and ASyS but low in IMNM [14].

The currently available evidence supports consideration of off‐label tofacitinib therapy in patients with IIM refractory to conventional treatments. Prospective controlled studies are, however, needed to conclusively establish the efficacy of JAKis in treating IIMs and to support their inclusion in treatment guidelines.

## Funding

This work received no specific grant from any funding agency in the public, commercial, or nonprofit sectors.

## Consent

All patients allowed personal data processing, and informed consent was obtained from all individual participants included in the study.

## Conflicts of Interest

The authors declare no conflicts of interest.

## Data Availability

The data that support the findings of this study are available on request from the corresponding author. The data are not publicly available due to privacy or ethical restrictions.
